# Round the Hospitals

**Published:** 1917-06-30

**Authors:** 


### ROUND THE HOSPITALS.

In accordance with her practice, Queen Alexandra on the morning of Rose Day received at Marlborough House the matrons, sisters, and staff nurses of the Military Nursing Services, who have been awarded the Royal Red Cross, subsequent to the investiture at Buckingham Palace. Her Majesty, accompanied by the Princess Victoria and attended by the Duchess of Portland (Mistress of the Robes), the Hon. Charlotte Knollys (Lady-in-Waiting), General the Right Hon. Sir Dighton Probyn (Comptroller), and Colonel Sir Henry Streatfeild (Equerry), afterwards drove through London to see the sale of roses which was being carried on in aid of the London hospitals and charitable institutions. Her Majesty was welcomed everywhere with enthusiasm, and her popularity with the people of all classes was as gratifying as it is well earned and deserved.

The *Weekly Irish Times* of the 23rd inst. publishes a very interesting and instructive letter from Miss Vera Matheson, Secretary to the Irish Board of the College of Nursing, Limited. She points out that "The analogy drawn by Miss Kearns between trades unions and the nursing profession deals with cases that are not analogous. Nursing is a profession, and all honourable professions aim at unity with the highest professional skill and keenness, a lofty aim, a broad outlook, and wide sympathies. Trained nurses, whilst endeavouring to ensure present material betterment, look also into the future, and seek to build their profession upon broad and honourable bases, which will gain for all branches of it a name honoured throughout the world." These have been the aims of the best British nurses of all ranks and of those who can best claim to be their real friends and helpers through the College of Nursing, Limited.

Miss Matheson further declares that nurses in Ireland who have thrown in their lot with the College of Nursing have done so in no petty spirit of partisanship, but because they believe in its loftiness of aim and breadth of outlook. Irish nurses look to the College to accomplish for the nursing profession those practical reforms which all desire, to give members of the College solid material advantages and at the same time to preserve that vision of the ideal "without which," as a Hebrew prophet says, "the people perisheth." Miss Matheson further and rightly insists that a good deal of nonsense is being talked just now in regard to nurses as recipients of charity. Eleemosynary gifts have benefited and are benefiting all classes in Great Britain, from the highest to the lowest. Moreover, members of every profession accept them, for each, including the medical and legal, has its Benevolent Fund and would be distinctly surprised were it suggested that such a fund was derogatory to the dignity of their profession. The rank and file of working nurses are not always able to put sufficient by for the ultimate rainy day; and when sickness comes to them they may speedily use up their hard-earned savings. Nurses often have others dependent upon them, and though many of them may not mind and often do starve themselves, there are few nurses, like the majority of the race, to their credit, who do not break down when those dependent upon them must starve too. Hence a great Benevolent Fund for nurses, similar to that of the legal, medical, and other professions, is an object which all human and self-respecting people would welcome and support with the assured certainty that this is an organisation which would be above criticism. Miss Matheson insists with truth on the unspeakable advantage it would be to nurses as a whole if all their differences could be sunk and every member of the profession work in concert for the advancement of the nursing profession, with its honourable traditions which all trained nurses are proud to uphold and share.

There are two points which matrons, sisters, and trained British nurses should clearly understand. First, that supporters of the Central Committee for the State Registration of Trained Nurses persist in the circulation of the erroneous statement that its "negotiations with the College Council were broken off by the Central Committee, because the College Council broke its agreement as to the constitution of the Provisional Council set up in the Bill, depriving the nurses of direct representation, and refused to consider the demand of the Central Committee that it should keep its bond." In fact, the Council of the College has kept its bond in that it never receded from its position that the nurses' societies working for the State Registration of Trained Nurses should have just as many nominees on the first Council as the College. The Central Committee, representing these societies, was actually asked to name these nominees (see The Hospital, November 18, 1916, pages 143-146). This it refused to do, although Mr. Stanley pointed out that the Committee's proposal to ask Parliament to entrust to bodies of whom it knew little or nothing the nomination of the members of the first General Nursing Council, was not likely to be viewed favourably by members of Parliament, and might wreck the Bill. So far from receding from the position Mr. Stanley had taken up all along in the conferences held, he insisted that the Central Committee should have just as many nominees on the first Council as the College had, which demonstrates the *bona fides* of the College Council, and exposes the hollowness of the claim now put forward by the Central Committee that it alone is composed of honourable people and pledged to its members to a certain course which the College first agreed to and then repudiated. The simple fact of the matter is that the Central Committee refused to stand by the agreement and thereupon broke off negotiations. If there has been repudiation the charge must therefore properly lie against the Central Committee, and not against the Council of the College.

The second point is that the representatives of the National Poor-Law Officers' Association have insisted that their help is necessary to protect the rights and claims of Poor-Law nurses from the policy of the College and others who have relegated Poor-Law nurses to a position of inferiority. In fact, nurses holding a certificate from a Poor-Law infirmary recognised as a training-school by the Local Government Board are eligible for membership of the College of Nursing under the conditions laid down for existing nurses applicable during the period of grace. It is not the desire of the College to differentiate between the Poor-Law-trained and the hospital-trained nurse. On the contrary, each of them is registered in exactly the same way, and their papers filed in the same cabinet. When the regulations for the membership of the College were drawn up the information received in reference to Poor-Law nurses was that the Local Government Board's standard for recognition of training-schools required 250 beds and a resident medical officer. Consequently this was the standard first adopted by the College. It was not until after these regulations had been printed that it was found that these conditions in some instances had been waived by the Local Government Board. The Council of the College, on making this discovery, added another clause to their regulations which admitted "all schools recognised at the present date (December 1916) by the Local Government Board as training-schools." This demonstrates that there is no difference whatever in the treatment extended by the College to the Poor-Law-trained and the hospital-trained nurse. Further, that the N.P.L.O.A. cry that the Poor-Law-trained nurses have been relegated to a position of inferiority is a pure myth having no foundation in fact.

Miss A. Rumsey, who for 12½ years has worked as inspector of the Q.V.J.I.N., Scottish Branch, and is well known throughout the whole of Scotland, having in addition performed the duties of assistant superintendent at the central offices at Edinburgh for the past 4½ years, has been offered the post of superintendent of the Scottish Branch of the Q.V.J.I.N. vacated by Miss Peterkin, and has accepted it for one year. Miss Rumsey has filled various posts in connection with the Institute in Scotland for over twenty-three years, and her promotion to the important office of superintendent for Scotland will give general satisfaction. Miss Rumsey was trained in general nursing at the County Hospital, Lincoln, and in district nursing in Edinburgh. Her record points to continuous success and great efficiency, and we hope the new and important position she has accepted will add to her laurels and the advancement of the Institute.

The National Baby Week promises to have a host of imitators up and down the country. Lady Jellicoe will open one at Guildford on July 10, when prizes are to be offered for the healthy and beautiful infants from three months to five years old. Lady Bray offers a special prize of 3s. in each class of babies to the winner, if vaccinated. The question of vaccination is to be excluded from consideration by the judges. The National Baby Week Council claim that the mortality among unvaccinated children is about 43.3 per thousand, and among the vaccinated only .09. The Council declare that by offering this prize Lady Bray is voicing the national need for vaccination among the babies and young children throughout the kingdom.

We have received the following notice: Members of the College of Nursing should keep the afternoon of Thursday, July 12, free for the First Annual Meeting of Registered Members of the College. Members will receive the official notice of the Meeting, Agenda, and Annual Report in the course of a few days. Accepted Candidates who have not yet paid their Fees of One Guinea should do so at once to entitle them to be present at their First Annual Meeting, which will be historical.

